# Comparative liver transcriptome analysis in ducklings infected with duck hepatitis A virus 3 (DHAV-3) at 12 and 48 hours post-infection through RNA-seq

**DOI:** 10.1186/s13567-018-0545-7

**Published:** 2018-06-20

**Authors:** Xuelian Zhang, Chong Cao, Yue Liu, Haihui Qi, Wenjing Zhang, Chunxue Hao, Haotian Chen, Qi Zhang, Wenlong Zhang, Mingchun Gao, Junwei Wang, Bo Ma

**Affiliations:** 1 Department of Preventive Veterinary Medicine, College of Veterinary Medicine, Northeast Agricultural University, Harbin, 150030 China; 2 College of Life Science and Engineering, Foshan University, Foshan, 528231 Guangdong Province China; 3 Northeastern Science Inspection Station, China Ministry of Agriculture Key Laboratory of Animal Pathogen Biology, Northeast Agricultural University, Harbin, 150030 China

## Abstract

Duck hepatitis A virus 3 (DHAV-3), the only member of the novel genus *Avihepatovirus*, in the family *Picornaviridae*, can cause significant economic losses for duck farms in China. Reports on the pathogenicity and the antiviral molecular mechanisms of the lethal DHAV-3 strain in ducklings are inadequate and remain poorly understood. We conducted global gene expression profiling and screened differentially expressed genes (DEG) of duckling liver tissues infected with lethal DHAV-3. There were 1643 DEG and 8979 DEG when compared with mock ducklings at 12 hours post-infection (hpi) and at 48 hpi, respectively. Gene pathway analysis of DEG highlighted mainly biological processes involved in metabolic pathways, host immune responses, and viral invasion. The results may provide valuable information for us to explore the pathogenicity of the virulent DHAV-3 strain and to improve our understanding of host–virus interactions.

**Electronic supplementary material:**

The online version of this article (10.1186/s13567-018-0545-7) contains supplementary material, which is available to authorized users.

## Introduction

Duck virus hepatitis (DVH) causes a highly fatal, contagious, and rapidly spreading viral infection in young ducklings, characterized primarily by liver necrosis, hemorrhage, and high mortality. DVH typically affects ducklings under approximately 4 weeks of age. The disease is caused by duck hepatitis A virus (DHAV), which is the only member of the novel genus *Avihepatovirus*, in the family *Picornaviridae* [1]. DHAV has been classified into three serotypes: DHAV-1 is the original serotype and is the most widespread and virulent [2], DHAV-2 was isolated in Taiwan [3, 4], and DHAV-3 is the recently described serotype that was isolated in South Korea and China [5, 6].

The interactions between virus and host are largely determined by the virulence of the pathogen and the host immune response, which may lead to changes in host gene expression [7]. DHAV can induce typical duckling liver lesions; however, many aspects of DHAV-3-host interactions remain unclear, and there is limited information available regarding gene expression changes in duckling liver cells in response to DHAV-3 infection.

The transcriptome is the sum of all gene transcription products of a specific tissue or cell in a functional state, and links the genetic information of the genome with proteomic biology functions. Transcriptomic studies are the basis of and provide a starting point in the study of gene function and regulatory networks. It is being increasingly recognized that transcriptome sequencing is an efficient means of characterizing the molecular basis of host–virus relationships [8, 9]. It also facilitates functional genomic studies, including profiling of global gene expression, assembly of full-length genes, and novel gene discovery. To date, many studies have reported on transcriptome sequencing in ducks. It is being widely used in the studies on duck-pathogens [10–15], duck-phenotypes [16, 17] and duck-performance interaction [18]. In 2013, Tang et al. carried out transcriptome sequencing to explore and compare the gene expression patterns of normal and DHAV-3 infected duckling livers at 24 hours post-infection (hpi) [10]. However, transcriptome sequencing at other time points of the duckling liver cells infected with DHAV-3 is absent.

In this study, transcriptome data generated using the livers of ducklings infected with lethal DHAV-3 at different infection time points was comparatively analyzed. The sequenced segments were compared and noted for screening of the differentially expressed immune-related genes using the GO and KEGG databases in NCBI. Some of the differentially expressed genes were verified by quantitative real-time PCR (qRT-PCR). This study may provide a foundation for future research on the understanding of the pathogenesis of DHAV-3 infection and may facilitate the discovery of the candidate genes that can respond to and resist DHAV-3 infection in ducks. Furthermore, it may extend the knowledge of the nature of virus–host interactions.

## Materials and methods

### Ethics statement

The study was approved by the Committee on the Animal Ethics of Northeast Agricultural University. Experiments were carried out in accordance with the approved guidelines.

### Virus and animals

The highly virulent DHAV-3 strain used in this study was isolated in the Guangdong Province. It was a gift from Prof. Guihong Zhang of the South China Agricultural University. Virus stocks were propagated in the allantoic cavities of 10-day-old specific-pathogen-free (SPF) embryonated duck eggs. The virus titer was determined to be a 10^6.375^ lethal median dose (DLD_50_)/0.2 mL and the calculation of it followed the Reed and Muench method. The virus was stored at −80 °C until further use.

Three-day-old SPF Jinding ducklings were obtained from the Laboratory Animal Center in the Harbin Veterinary Research Institute at the Chinese Academy of Agricultural Sciences (HVRI; Harbin, China) and housed in isolators until use. All animal experiments were performed according to the guidelines of the Committee on the Ethics of Animals of Heilongjiang and the appropriate biosecurity guidelines.

### Animal experiments

In this study, 20 5-day-old ducklings were randomly chosen and inoculated intramuscularly with 0.2 mL of lethal DHAV-3 under 10^5^ dilutions. We chose three ducklings to explore transcriptome sequencing at 0, 12, and 48 hpi.

### Sample collection and preparation

At 0, 12, and 48 hpi, three ducklings with DHAV-3 infection were euthanized and necropsied, and fresh liver tissue samples were collected from the animals. The samples for the transcriptomics analysis were rapidly placed in liquid nitrogen and the others were stored at −70 °C for further experiments until viral titration. Parts of the tissues were fixed with 10% formaldehyde in PBS for histopathological examination. The ducklings’ heart, liver, spleen, lung, kidney, large intestine, small intestine, brain, thymus, bursa of fabricius, and muscle were also collected. Clinical symptoms and the results of the autopsy were recorded.

### RNA quantification and qualification

RNA degradation and contamination was monitored on 1% agarose gels. RNA purity was checked using the NanoPhotometer^®^ spectrophotometer (IMPLEN, CA, USA). RNA concentration was measured using Qubit^®^ RNA Assay Kit in Qubit^®^ 2.0 Flurometer (Life Technologies, CA, USA). RNA integrity was assessed using the RNA Nano 6000 Assay Kit of the Bioanalyzer 2100 system (Agilent Technologies, CA, USA) for further cDNA synthesis and sequencing. RIN (RNA Integrity Number) threshold reached more than eight in the present study, which proved that RNA integrity was perfect.

### Library preparation for transcriptome sequencing

Nine duckling liver samples were collected and stored in liquid nitrogen; total RNA extraction and quality testing was done and nine cDNA libraries were created. RNA (3 µg/sample) was used as input material for the RNA sample preparations. Sequencing libraries were generated using NEBNext^®^ Ultra™ RNA Library Prep Kit for Illumina^®^ (NEB, USA) according to the manufacturer’s recommendations, and index codes were added to attribute sequences to each sample. Briefly, mRNA was purified from total RNA using poly-T oligo-attached magnetic beads. Fragmentation was carried out using divalent cations under elevated temperature in NEBNext First Strand Synthesis Reaction Buffer (5×). First strand cDNA was synthesized using a random hexamer primer and M-MLV Reverse Transcriptase (RNase H^−^). Second strand cDNA synthesis was subsequently performed using DNA polymerase I and RNase H. Remaining overhangs were converted to blunt ends via exonuclease/polymerase activities. After adenylation of the 3′ ends of the DNA fragments, NEBNext adaptor with a hairpin loop structure were ligated to prepare for hybridization. In order to select cDNA fragments of preferentially 150–200 bp lengths, the library fragments were purified with the AMPure XP system (Beckman Coulter, Beverly, USA). Then 3 µL USER Enzyme (NEB, USA) was used with size-selected, adaptor-ligated cDNA at 37 °C for 15 min followed by 5 min at 95 °C before PCR. Next, PCR was performed with Phusion High-Fidelity DNA polymerase, Universal PCR primers, and Index (X) Primer. Finally, PCR products were purified (AMPure XP system) and library quality was assessed on the Agilent Bioanalyzer 2100 system.

### Clustering and sequencing

The clustering of the index-coded samples was performed on a cBot Cluster Generation System using TruSeq PE Cluster Kit v3-cBot-HS (Illumia) according to the manufacturer’s instructions. After cluster generation, the library preparations were sequenced on an Illumina Hiseq platform and 125 bp/150 bp paired-end reads were generated.

### Data analysis

#### Quality control

Fastq format raw data (raw reads) were first processed through in-house perl scripts. In this step, clean data (clean reads) were obtained by removing reads containing adapters, reads containing ploy-N, and low-quality reads from raw data. At the same time, Q20, Q30, and GC content in the clean data were calculated. All the downstream analyses were based on the high-quality clean data.

#### Reads mapping to the reference genome

Reference duck genome (BGI_duck_1.0) and gene model annotation files were downloaded from the National Center for Biotechnology Information (NCBI) [19]. The index of the reference genome was built using Bowtie v2.2.3 and paired-end clean reads were aligned to the reference genome using TopHat v2.0.12. We selected TopHat as the mapping tool since TopHat can generate a database of splice junctions based on the gene model annotation file, and thus, provide better mapping results than other non-splice mapping tools.

#### Quantification of gene expression level

HTSeq v0.6.1 was used to count the read numbers mapped to each gene. Then the FPKM of each gene was calculated based on the length of the gene and read counts were subsequently mapped. FPKM, an expected number of fragments per kilobase of transcript sequence per million base pairs sequenced, considers the effect of sequencing depth and gene length for the read counts at the same time, and is currently the most commonly used method for estimating gene expression levels [20].

#### Differential expression analysis

Differential expression analysis of two conditions/groups (two biological replicates per condition) was performed using the DESeq R package (1.18.0). DESeq provides statistical routines for determining differential expression in digital gene expression data using a model based on the negative binomial distribution. The resulting *P*-values were adjusted using the Benjamini and Hochberg approach for controlling the false discovery rate. Genes with an adjusted *P*-value < 0.05 found by DESeq were considered differentially expressed.

#### GO and KEGG enrichment analysis of differentially expressed genes

Gene ontology (GO) enrichment analysis of differentially expressed genes was implemented by the GOseq R package, in which gene length bias was corrected. GO terms with corrected *P*-value < 0.05 were considered significantly enriched by differentially expressed genes.

KEGG is a database resource for understanding high-level functions and utilities of a biological system, such as a cell, organism, or ecosystem, from molecular-level information, especially large-scale molecular datasets generated by genome sequencing and other high throughput experimental technologies. We used KOBAS software to test the statistical enrichment of differentially expression genes in KEGG pathways.

#### qRT-PCR for confirmation

Total RNA of the collected tissues were extracted with TRIzol Reagent (Invitrogen, Carlsbad, CA), according to the manufacturer’s instructions. Each RNA sample was reverse-transcribed with PrimeScript™ RT reagent Kit with gDNA Eraser (Takara, Dalian, China) according to the manufacturer’s instructions. The concentration of the synthesized cDNA was measured using an ND-1000 Spectrophotometer (Nanodrop Technologies, Wilmington, DE) and the cDNA was stored at −20 °C until analysis.

Fourteen immune related genes were selected for confirmation and the primers used for qRT-PCR assays are listed in Additional file 1. qRT-PCR was performed in a reaction volume of 20 μL with the 7500 Real-Time PCR System (Applied Biosystems, Carlsbad, CA, USA) using the SYBR Green PCR kit (Takara, Dalian, China) according to the manufacturer’s instructions. All primer pairs were selected according to their specificity, determined with dissociation curves. The PCR cycling conditions were the following: one cycle at 95 °C for 30 s, 40 cycles of denaturation at 95 °C for 5 s and extension at 60 °C for 34 s, followed by a dissociation curve analysis step. To validate the assay, the purified PCR products were cloned into the pMD18-T plasmid and sequenced to confirm proper amplification. Each sample was analyzed in triplicate.

### Statistical analysis of qRT-PCR

The relative expression of the target genes in the infected group and in the control group was calculated with the 2^−∆∆Ct^ method and quantified relative to the housekeeping gene encoding beta-actin (β-actin), which was employed as the endogenous control to normalize the expression levels of the target genes, then expressed as the fold change in gene expression.

## Results

### Gross lesions, histopathological analysis, and virus loads of DHAV-3-infected ducklings

**Figure 1:**
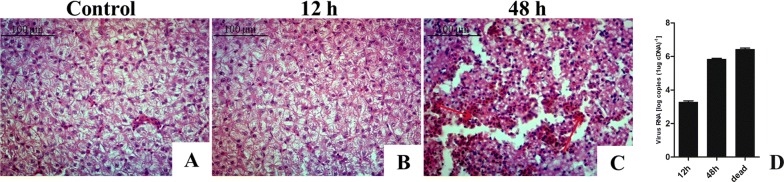
**Pathological changes in the livers of DHAV-3-infected ducklings at different time points. A** Histopathological examination of duckling livers in the control group. **B** Histopathological changes were not observed in the liver at 12 hpi. **C** Many cells have dissolved and disappeared, hepatocyte necrosis with hemorrhage at 48 hpi. **D** DHAV-3 replication in the livers of ducklings with DHAV-3 infection at 12, 48, and 72 hpi. The data are expressed as mean ± standard deviation. Three dead ducklings were selected for detecting the viral RNA load using the qRT-PCR method.

Ducklings infected with virulent DHAV-3 showed neurological symptoms as early as approximately 72 hpi and all ducklings were dead by then. Anatomical analysis revealed that infected 5-day-old ducklings had typically visibly-enlarged petechial and ecchymosis hemorrhages throughout the liver at 48 hpi, but no visible lesions at 12 hpi. Histological analysis of the liver specimens revealed significant differences between virulent DHAV-3 infected ducklings exhibiting a systematic cell swelling and vesicular degeneration (hydropic degeneration) at 48 hpi (Figure 1C), but no significant histopathological differences were observed in the livers between the control (Figure 1A) and infection groups at 12 hpi (Figure 1B). We detected DHAV-3 loads in the livers using qRT-PCR. DHAV-3 replicated rapidly in the liver and reached 10^3.21^ copies (1 μg cDNA)^−1^ at 12 hpi, 10^5.84^ copies (1 μg cDNA)^−1^ at 48 hpi, and 10^6.42^ copies (1 μg cDNA)^−1^ in the livers of the dead ducklings (Figure 1D). The viral titer was detected in 11 tissues (heart, liver, spleen, lung, kidney, large intestine, small intestine, brain, thymus, bursa of fabricius, and muscle) and the highest titer was observed in the liver (data not shown).

### Construction of cDNA libraries and DEG after lethal DHAV-3 infection

Using Illumina Hiseq platform, 457 million (457 766 570) raw reads were produced. To guarantee the ideal results for genome mapping and differential gene change analysis, raw reads were filtered to remove low quality data, resulting in a total of 437 million (437 610 926) clean reads (Additional file 2).

**Figure 2:**
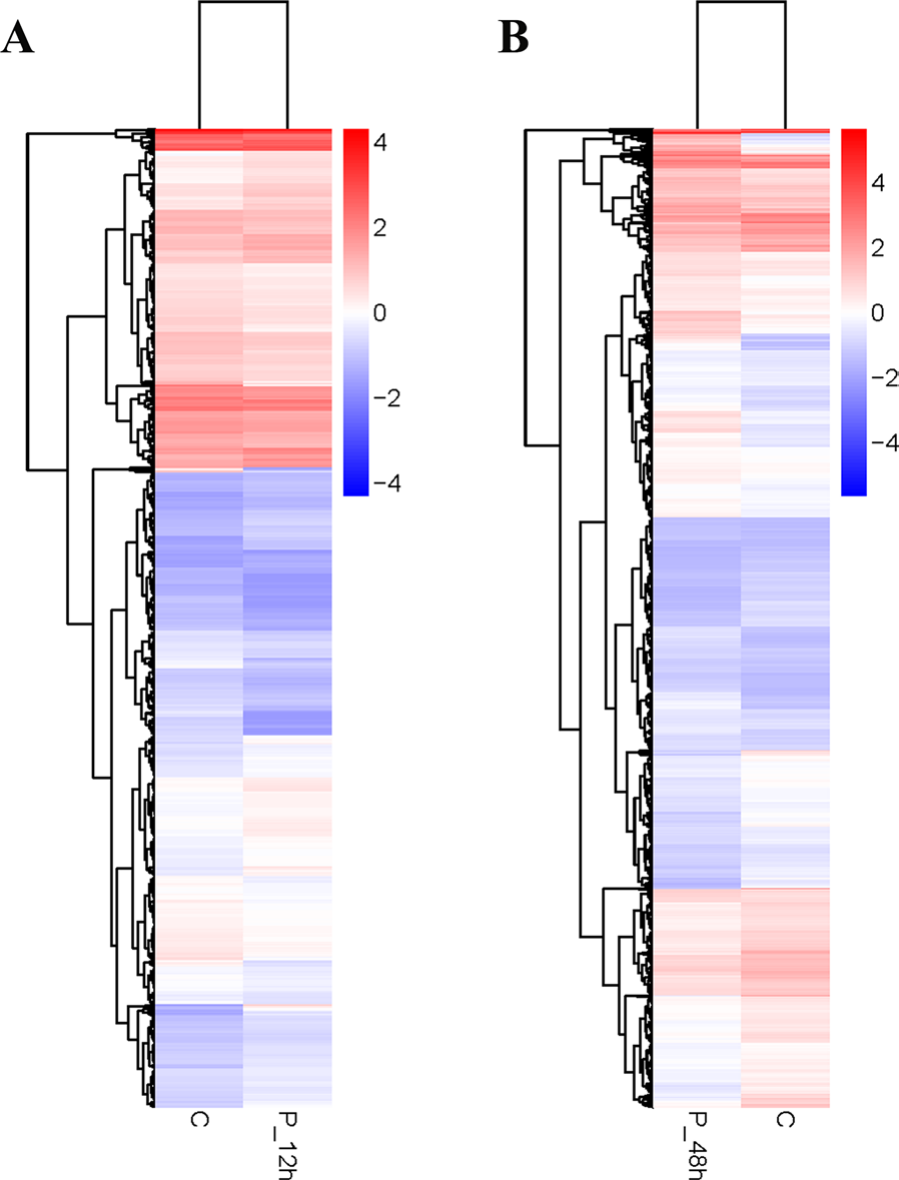
**Heat map analysis is used to classify gene expression patterns at 12 hpi (A) and 48 hpi (B).** Genes with similar expression patterns were clustered, as shown in the heat map. Intensity of color indicates gene expression levels that were normalized according to log10 (FPKM + 1) values. Red represents high expression level genes and blue represents low expression level genes.

**Figure 3:**
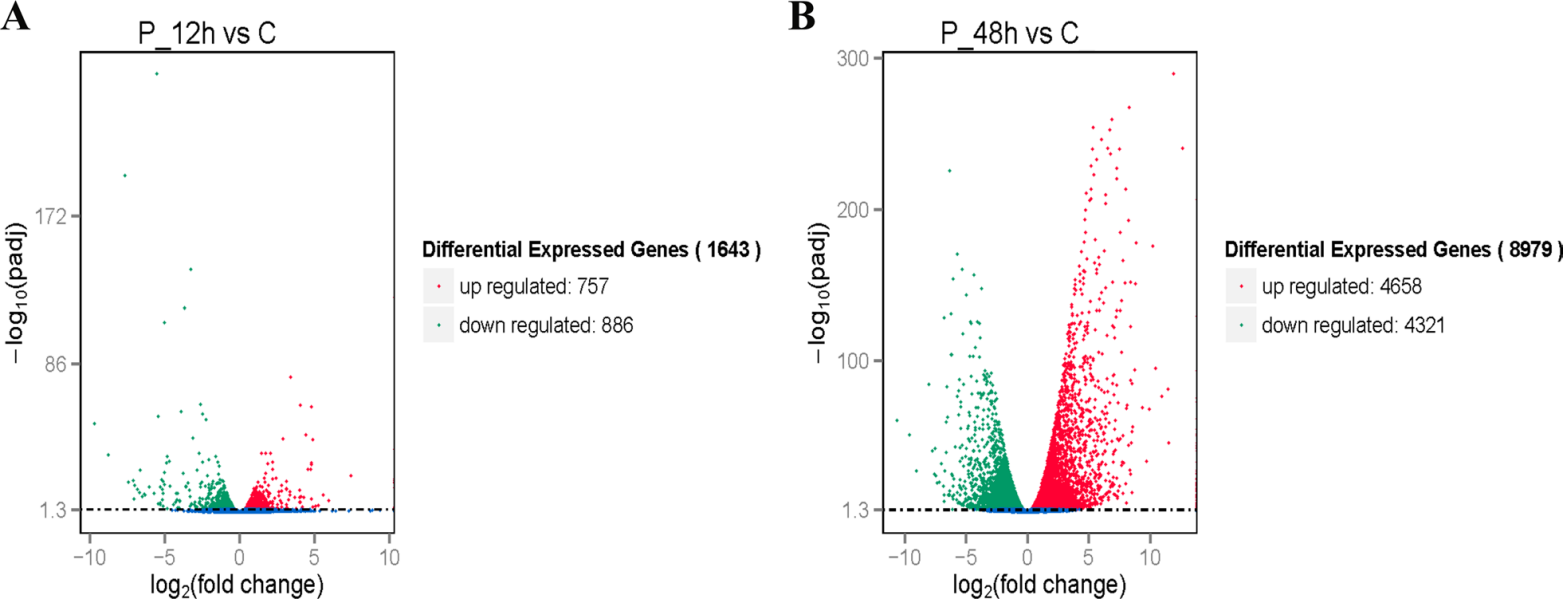
**Volcano plots of differentially expressed genes in the livers of the ducklings at 12 hpi (A) and 48 hpi (B).** Red points represent up-regulated genes, green points represent down-regulated genes, and blue points represent genes with no significant difference.

A heat map visually compares all differentially expressed genes (DEG) and classifies gene expression patterns according to different time points (Figures 2A and B). In order to obtain a global view of the change in duck gene expression between the different experimental groups at different time points, two paired comparisons (12 hpi vs control, 48 hpi vs control) were performed. In all, RNA-seq analysis detected 1643 and 8979 genes, which were expressed at significantly different levels in the 12 and 48 hpi DHAV-3 animals, respectively, compared with the control at a *P*-value < 0.05 (Figures 3A and B). DHAV-3 infection contributed to the differential expression of 757 genes that were up-regulated and 886 that were down-regulated in the liver compared with those in the control ducklings at 12 hpi, and 4658 that were up-regulated and 4321 that were down-regulated at 48 hpi. At 48 hpi, DEG are associated with immune and inflammatory responses and may play important roles in the host defense response to DHAV-3 infection.

**Figure 4:**
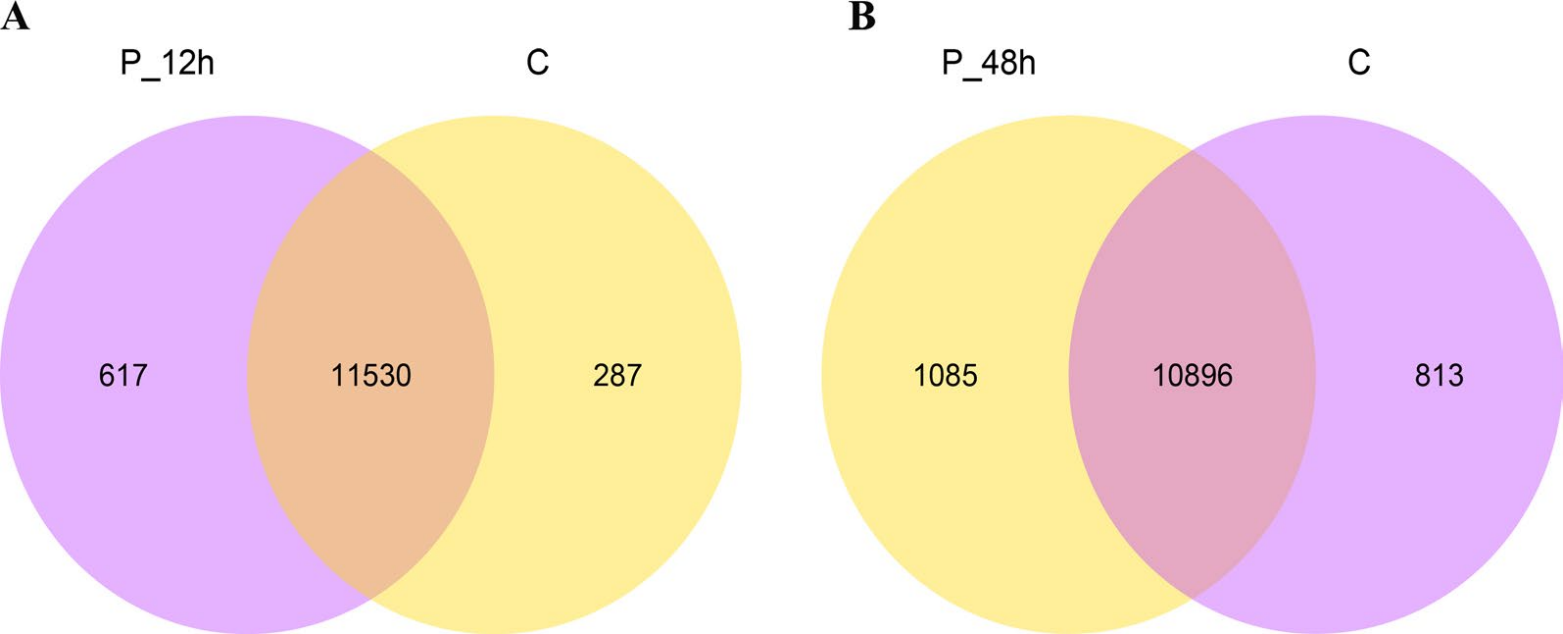
**Venn diagram displaying a global view of the numbers of differentially expressed genes.** The overlap of differentially expressed genes at 12 hpi vs control **(A)** and 48 hpi vs control **(B)**. The numbers in the diagram indicate gene number and refer to each comparison.

In order to more precisely examine the differentially expressed genes, Venn analysis was utilized (Figures 4A and B) and there were three main parts of each diagram. There were 617 and 1085 genes representing the numbers of genes only expressed in the DHAV-3 infected ducklings at 12 and 48 hpi, respectively, when compared with those in the control. The list of all differentially expressed genes at 12 hpi compared with those in the control group are displayed in Additional file 3. The list of all differentially expressed genes at 48 hpi compared with those in the control group are displayed in Additional file 4. Compared with the control group, genes expressed only in the livers of the 12 hpi ducklings are displayed in Additional file 5 and those in 48 hpi ducklings are displayed in Additional file 6.

### Annotation of duckling liver DEG based on GO analysis after DHAV-3 infection

To ensure differentially expressed gene function, gene ontology (GO) analysis was performed to categorize and annotate DEG into three groups, including biological processes (BP), cellular components (CC), and molecular function (MF).

**Figure 5:**
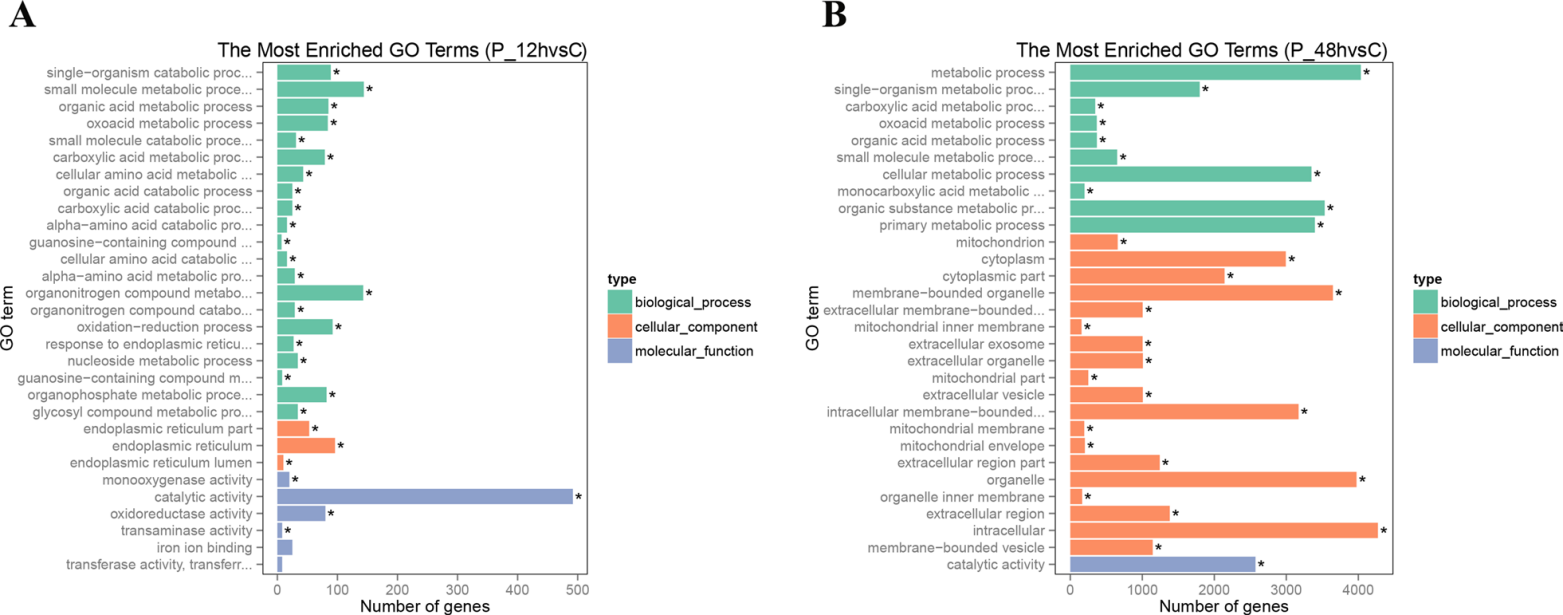
**Top 30 gene ontology (GO) terms of DEG expressed at 12 and 48** **hpi.** GO-terms were processed under three categories including cellular component (CC), molecular function (MF), and biological process (BP). The top 30 GO terms were selected according to *P*-value < 0.05. **A** GO annotation of DEG expressed at 12 hpi. **B** GO annotation of DEG expressed at 48 hpi.

In order to pick out the helpful and useful genes for further exploration, 30 significant GO terms were listed. The top 30 GO terms were selected according to *P*-value < 0.05. The top three significant GO terms of DEG expressed in DHAV-3 infected at 12 hpi were catalytic activity (GO: 0003824), small molecule metabolic process (GO: 0044282), and organonitrogen compound metabolic process (GO: 1901565) (Figure 5A). The top three significant GO terms of DEG expressed in DHAV-3 infected at 48 hpi were intracellular (GO: 0005622), metabolic process (GO: 0008152), and organelle (GO: 0043226) (Figure 5B). The description of all genes in the livers of ducklings at 12 and 48 hpi are displayed in Additional files 7 and 8. The lists contain the structure of the genes, expression level of the genes, different information for each gene, and annotation information. In addition, the lists of GO annotations for genes in the livers of the 12 and 48 hpi ducklings in this study are displayed in Additional files 9 and 10.

### Pathway analysis of DEG based on KEGG after DHAV-3 infection

**Figure 6:**
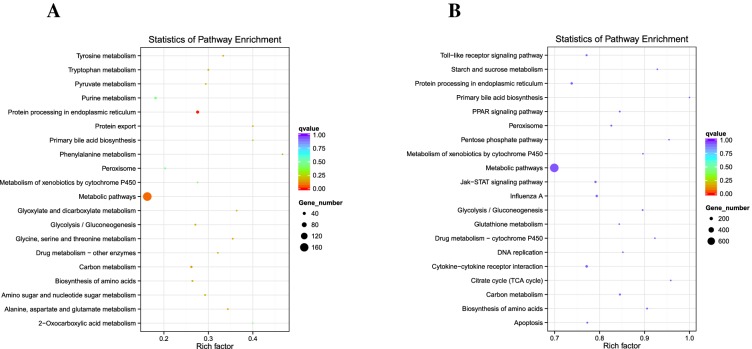
**Top 20 KEGG pathways at 12 hpi and 48 hpi. A** KEGG pathways of DEG at 12 hpi. **B** KEGG pathways of DEG at 48 hpi.

The KEGG database was used to analyze pathways in order to further define DEG function in duckling livers after DHAV-3 infection. The top 20 enrichment KEGG pathways are listed in Figure 6 according to *P*-value < 0.05. Three functional categories were identified to potentially play important roles associated with DHAV-3 at 12 hpi infection, including metabolic pathways, protein processing in endoplasmic reticulum, and phenylalanine metabolism. Four functional categories were identified to potentially play important roles associated with DHAV-3 at 48 hpi infection, including metabolic pathways, cytokine–cytokine receptor interaction, Jak-STAT signaling pathway, and Toll-like receptor signaling pathway. Primers associated with the immune pathway analysis of 48 hpi of DHAV-3 infected ducklings are displayed in Additional file 1. The putative functional roles and interactions of these genes involved in mediating the host response of ducklings are discussed in detail (Table 1).

**Table 1:** **Associated with the immune pathway in liver transcriptome of ducklings infected with DHAV-3 at 48 hpi**

Description	*P*-value	Corrected *P*-value	Number of DEG
Cytokine–cytokine receptor interaction	2.49E−07	3.76E−05	98
Influenza A	3.93E−05	0.001483228	75
Herpes simplex infection	0.002300281	0.043417795	75
Jak-STAT signaling pathway	3.76E−05	0.001483228	68
Toll-like receptor signaling pathway	3.17E−05	0.001483228	56
Apoptosis	0.001142807	0.028760647	41
Focal adhesion	0.000688445	0.020791028	35
RIG-I-like receptor signaling pathway	0.001372704	0.029611189	31

### Verification of DEG by qRT-PCR

In order to further confirm the differential gene expression obtained from the transcriptome sequencing data, we analyzed by qRT-PCR the expression levels of 14 kinds of immune related genes that were mainly involved in host immune defense responses against DHAV-3 infection, including TLR7, TLR3, RIG-1, MDA5, IFN-α, IFN-β, IFN-γ, IL-1β, IL-2, IL-6, MX, OAS, PKR, and IFIT5 at 48 hpi. The results show that only IL-1β was not detected in the ducklings infected at 48 hpi, and the expression patterns of the remaining genes were correlated with the transcriptome data (Table 2), indicating the reliability of the transcriptome sequencing data.

**Table 2:** **Verification of real-time PCR for some differentially expressed genes**

Genes	Transcriptomics fold change (infected/control)	qRT-PCR fold change (2^−∆∆Ct^) (infected/control)	Gene function
RIG-1	21.3	6.69	RIG-like receptor
MDA5	37.3	31.48	RIG-like receptor
TLR7	6.429	25.3	Toll-like receptor
TLR3	4.564	4.23	Toll-like receptor
IFN-α	Inf	160.9	Type I IFN
IFN-β	226.29	702.86	Type I IFN
IFN-γ	7.66	5.63	Type II IFN
IL-1β	–	78.09	Proinflammatory cytokines
IL-2	Inf	171.88	Proinflammatory cytokines
IL-6	31.16	68.62	Proinflammatory cytokines
MX	1464	3880.75	IFN-stimulated genes
OAS	442.74	191.77	IFN-stimulated genes
PKR	31.25	19.85	IFN-stimulated genes
IFIT5	4110.1	2103.33	IFN-stimulated genes

“Inf” represented the differential expression is infinite is in the results of transcriptome sequencing.

“–” represented the gene did not detect through RNAseq.

## Discussion

Duck virus hepatitis, mainly caused by DHAV, is a severe threat to the duck industry all over the world. Previous studies of DHAV have focused only on the pathogenicity and host response of DHAV-1, while relatively few reports have focused on DHAV-3. In this study, the pathological changes induced by DHAV-3 and DHAV-1 were similar. The principle lesions caused by DHAV-3 were found in the liver, which contained punctate and ecchymosis hemorrhages. Significantly more viral RNA copies were detected in the liver than in other organs, also suggesting that the main target organ is the liver. Moreover, the viral loads of each sampling in the liver detected from 48 hpi were significantly higher than those from 12 hpi, and the lesions in the liver of ducklings at 48 hpi were more serious than those at 12 hpi (Figure 1). The results show that with the increased proliferation of DHAV-3 in the liver, the damage of liver parenchyma cells was increased.

RNA-seq technology is a high-throughput sequencing technology that can reveal the molecular mechanisms, biological processes, and development of disease. So far, many reports have analyzed transcriptome sequences of different tissues in DHAV-, reovirus-, and DHBV-infected ducks [11–13], providing basic data for understanding the molecular mechanism of viral infections in ducks.

In the present study, in order to describe the genetic architecture of duckling liver transcriptomes and to further facilitate investigations on the molecular events during DHAV-3 infection, we performed transcriptome analysis on normal ducklings (0 hpi), and on ducklings at 12 and 48 hpi following infection with a highly pathogenic DHAV-3 strain.

After removal of low-quality sequences and assembly, we obtained 437 million clean reads. To ensure differentially expressed gene function, GO and KEGG signaling pathway analyses were performed to categorize and annotate DEG. By comparing the transcriptome data of the three libraries, the results suggest that most DEG were associated with metabolic pathways and amino acid metabolism at 12 hpi. DEG were also associated with metabolic pathways, apoptosis, and immune genes related to signaling pathways at 48 hpi. The regulation of actin cytoskeleton (84 genes) and FoxO signaling pathways (59 genes) was affected at 48 hpi. The prompted host did not clear the virus effectively, and the virus continued to affect host cell growth and differentiation. These data can provide useful information for further investigation into the process of DHAV-3 infection in ducklings.

The study by Zhang used RNA-seq to analyze liver transcriptomes for dying ducklings and the results suggested that DHAV-1 infection could down-regulate some gene expression of related metabolic pathways and inhibit the metabolism of the host cell, as well as up-regulate the expression of immune genes to inhibit the replication of virus infection [15]. We speculate that the immune response mechanism of DHAV-3-infected ducklings is similar to that of DHAV-1 infection, in that DHAV-3 infection first affected the metabolism of liver cells, which caused disorders in duckling livers and created favorable conditions for virus replication in the host. In conjunction with the increase in virus copy numbers in the liver, the host mobilized many immune related genes for resistance to viral infection, but ultimately failed to resist infection, leading to the dysfunction of most organs and eventual death.

The study by Zhang showed that DHAV-3 infections induce host cell apoptosis and necrosis of multiple organs, including the liver, which is an important means of host resistance to the virus, but the mechanism is not understood [21]. In the present study, the expression of 41 DEG in apoptosis signaling pathways was up-regulated at 48 hpi, which was consistent with the results in the study by Zhang. Tang et al. conducted RNA-seq of DHAV-C infection on ducklings at 24 hpi. The results show that TLR, NLR, and RIG-I-like receptors were not significantly up-regulated, consistent with the RNA-seq results of duckling livers at 12 hpi in the present study [10]. We hypothesize that DHAV-3 has a mechanism of immune evasion, which prevents the significant activation of the immune genes in the early stages of infection, but this may also depend on the virulence of the virus and the age of the ducks.

KEGG signaling pathway annotations to DEG showed that the expression of immune response related genes at 48 hpi is mainly involved in cytokine–cytokine receptor interaction, the Jak-STAT signaling pathway, Toll-like receptor signaling pathway, and RIG-I receptor signaling pathways. We verified the relative expression of 14 kinds of immune related genes by qRT-PCR. The results show that, except for IL-1β, the expression of the other genes was consistent with the transcriptome sequencing results. DESeq sequencing software was used to treat the large amount of data obtained. For the qRT-PCR data, gene expression was calculated with the formula 2^−∆∆Ct^. Therefore, it is reasonable to have some inconsistency between RNA-seq and qRT-PCR. Combining the results of RNA-seq and qRT-PCR, MDA5, TLR7, IFN-α, IFN-β, IL-2, IL-6, MX, OAS, and IFIT5 may play more important roles in understanding the process of duckling resistance to DHAV-3 infection. In future research, we will focus on the above genes.

Innate immunity provides a first line of defense against pathogens and can be rapidly activated following infection. Activation of the innate immune system relies on the recognition of pathogen-associated molecular patterns (PAMP) by specific pattern-recognition receptors (PRR). TLR play a role in recognizing viruses in mammals. TLR are highly activated in acute inflammatory responses to pathogens. In the Toll-like receptor signaling pathway, the expression of TLR2, TLR3, TLR4, TLR5, TLR7/8 were all significantly up-regulated at 48 hpi. dsRNA derived from the viruses has been shown to bind to TLR3 and activate the nuclear factor NF-κB pathway, which leads to the production of type I IFN, including IFN-α and IFN-β. In ducks, TLR7/8 could recognize ssRNA, triggering TLR7 to rapidly up-regulate pro-inflammatory cytokines and IFN-α, critical mediators of antiviral defense. TLR7 and TLR3 were significantly up-regulated at 36 hpi in 3-week-old ducks infected with DHAV-1, in agreement with the results of the present study, suggesting that TLR7 and TLR3 possibly have a similar effect in sensing and initiating responses to DHAV infection.

MDA5 belongs to the RIG family of PRR and senses viral RNA in the cytoplasm. In this study, MDA5 expression levels were significantly increased after DHAV-3 infection. MDA5 could detect the double-stranded RNA replicative form in picornavirus-infected cells [22]. The report from Wang et al. showed that the expression levels of MDA5 in susceptible ducks were significantly higher than those in resistant ducks at 12 and 24 hpi [11], suggesting a role of MDA5 against DHAV-3 infection. We will detect other PRR for DHAV-3 recognition in future research.

DHAV can induce acute IFN-α and IFNβ production. The ability to induce IFNs is linked to the virulence and adaptability of the DHAV strain in a particular host system [23, 24]. In this study, type I IFN (IFNα and IFNβ) expression was significantly up-regulated after DHAV-3 infection. For example, IRF7, NF-κB, STAT1/2, MyD88, MX, OAS, PKR, and IFIT5 were all up-regulated. Therefore, we hypothesize that IFN might be induced during early infection, as indicated by the subsequent enhanced production of ISG. These pathways might exert their effects through different mechanisms of action, such as direct targeting of viral entry, inhibition of protein synthesis, or degradation of viral RNA, and play an important role in anti-DHAV-3 responses and host defense during DHAV-3 infection.

Cytokines play crucial roles in many biological cell processes and engage in humoral and cell-based immune responses, cell growth, differentiation, maturation, death, angiogenesis, and homeostasis. Through binding with corresponding cytokine receptors, cytokines transmit signals from outside into the cells. In our results, from the cytokine–cytokine receptor in the KEGG pathway, we found that the expression of many important cytokines and cytokine receptor genes were changed. Viral infections induce a pro-inflammatory response that includes cytokine and chemokine expression [25]. In the present study, increased expression of IL-2, IL-6, IL-10, IL-10RA, IL-17RA, CXCL12, CXCR4, CXCR6, CXCR7, CCL3, CCL4, CCL5, CCL20, and CCL28 was observed after DHAV-3 infection. IL-2 and IL-6 were also found to be significantly up-regulated in other viral infections in the duck, such as in infections with AIV, DTMUV, and DEV [14, 26, 27].

We obtained the transcription sequencing data from the livers of the ducklings infected with lethal DHAV-3 through RNA-seq technology at 12 and 48 hpi, respectively. The global profile of gene expression in the livers provided a good overview of the host response to DHAV-3 infection, which is helpful for understanding DHAV-3 mechanisms of disease or death. The results also provide basic information on the nature of virus-duckling interactions. Given the consistent utilization of current vaccines and the continued emergence of new virulent DHAV-3 genotypes throughout the world, the development of better vaccines and control strategies will require a greater understanding of the pathogenic mechanisms. However, analysis of the innate host immune response in the livers at two time-points may not be sufficient to completely understand the mechanisms of DHAV-3 pathogenesis. We hold the opinion that DHAV-3 infection seriously affects metabolic processes in the liver and immune related signal transduction in ducklings, but the findings from the analyses of molecular mechanisms and process of host immune response should be combined with the findings from transcriptome sequence analysis at varied time points, serum biochemical indexes, virus positioning, and histopathological observation for further elucidation. Therefore, further analysis of the above-mentioned concerns is warranted.

We screened and identified differentially expressed transcripts in DHAV-3-infected duckling liver tissues using RNA-seq. They were associated with immune and inflammatory responses, as well as a cytokine-mediated signaling pathway. Therefore, these genes may play major roles in the host defense response and/or DHAV-3 pathogenesis. Combining network analysis with differential gene expression, we identified that MDA5, TLR7, IFN-α, IFN-β, IL-2, IL-6, MX, OAS, and IFIT5 have potentially important roles for ducklings to defend against DHAV-3 infection. Our results provide comprehensive knowledge regarding the host transcriptional response to DHAV-3 infection in duckling liver tissues, thereby providing insight into DHAV-3 pathogenesis, particularly the involvement of innate immune pathway genes associated with DHAV-3 infection.

## Additional files

**Additional file 1.** **Primers used for qRT-PCR assays.** Fourteen immune related genes were selected for confirmation. The primers used for qRT-PCR assays are listed.

**Additional file 2.** **Number of reads of all bases detected using RNA-seq in DHAV-3-infected and control ducks.** To guarantee ideal results for genomic mapping and differential gene change analysis, raw reads were filtered to remove low quality data with a total of 437 million (437 610 926) clean reads acquired.

**Additional file 3.** **Differentially expressed genes in the uninfected and 12** **hpi duckling livers.** The list of all differentially expressed genes in the livers of ducklings in the control group and 12 hpi group.

**Additional file 4.** **Differentially expressed genes in the uninfected and 48** **hpi duckling livers.** The list of all differentially expressed genes in the livers of ducklings in the control group and 48 hpi group.

**Additional file 5.** **Genes expressed only in the livers of ducklings at 12** **hpi**. Compared with the control group, genes expressed only in the livers of ducklings at 12 hpi.

**Additional file 6.** **Genes expressed only in the livers of ducklings at 48** **hpi.** Compared with the control group, genes expressed only in the livers of ducklings at 48 hpi.

**Additional file 7.** **Description of all genes in the livers of ducklings at 12** **hpi.** Structure, expression level, difference information, and annotation information for all genes at 12 hpi are described.

**Additional file 8.** **Description of all genes in the livers of ducklings at 48** **hpi.** Structure, expression level, difference information, and annotation information for all genes at 48 hpi are described.

**Additional file 9.** **GO annotation information of genes in the livers of ducklings at 12** **hpi.**

**Additional file 10.** **GO annotation information of genes in the livers of ducklings at 48** **hpi.**

## Abbreviations

DHAV-3: duck hepatitis A virus type 3
DVH: duck virus hepatitis
DEG: differential expression genes
GO: gene ontology
KEGG: Kyoto Encyclopedia of genes and genomes
SPF: specific pathogen free
LD_50_: lethal median dose
hpi: hours post-infection
qRT-PCR: quantitative real-time RT-PCR
PRR: pattern recognition receptors
PAMP: pathogen associated molecular patterns
TLR: Toll-like receptors
RIG-1: retinoic acid inducible gene 1 protein
MDA5: melanoma differentiation-associated gene-5
TLR7: Toll-like receptor 7
TLR3: Toll-like receptor 3
IFN-α: interferon-α
IFN-β: interferon-β
IFN-γ: interferon-γ
IL-1β: interleukine-1 beta
IL-2: interleukin-2
IL-6: interleukin-6
MX: myxovirus-resistant
OAS: 2′–5′oligoadenylate synthetase
PKR: protein kinase R
IFIT5: interferon induced protein with tetratricopeptide repeats 5
ISG: IFN-stimulated genes
AIV: avian influenza virus
DTMUV: duck Tembusu virus
dsRNA: double-stranded RNA
ssRNA: single-stranded RNA
IRF7: interferon regulatory factor 7
NF-κB: nuclear transcription factor-κB
STAT1/2: signal transducer and activator of transcription ½
MyD88: myeloid differentiation primary response 88
IL-10RA/IL-17RA: interleukin 10/17 receptor alpha
CXCL12: chemokine (C-X-C motif) ligand
CXCR4/6/7: C-X-C chemokine receptor type 4/6/7
CCL3/4/5/20/28: chemokine (C-C motif) ligand 3/4/5/20/28
