# Supplementary material for: Comparative liver transcriptome analysis in ducklings infected with duck hepatitis A virus 3 (DHAV-3) at 12 and 48 hours post-infection through RNA-seq
Source: Vet Res. 2018 Jun 20;49:52. doi: 10.1186/s13567-018-0545-7 (PMC6011267; doi:10.1186/s13567-018-0545-7)
Supplement: Supplementary file 1 — Additional file 1. Primers used for qRT-PCR assays. Fourteen immune related genes were selected for confirmation. The primers used for qRT-PCR assays are listed. [file 13567_2018_545_MOESM1_ESM.docx]

| Target gene | Sequence (5′-3′) | length (bp) |
| --- | --- | --- |
| RIG-1-S | TTGGCAAACCTCCACCTGTC | 188 |
| RIG-1-A | AACTTTCGCCTTTCGTCCTG |  |
| MDA5-S | GCTACAGAAGATAGAAGTGTCA | 120 |
| MDA5-A | CAGGATCAGATCTGGTTCAG |  |
| TLR3-S | GAGTTTCACACAGGATGTTTAC | 200 |
| TLR3-A | GTGAGATTTGTTCCTTGCAG |  |
| TLR7-S | GTGGCAGCTTCAAGACAACA | 111 |
| TLR7-A | CATACCGGGACTTCTGCAAT |  |
| IFN-α-S | CCTCCTGGACACCAATGACA | 132 |
| IFN-α-A | TTGAGGAGGTCGTGGCGTG |  |
| IFN-β-S | AGATGGCTCCCAGCTCTACA | 210 |
| IFN-β-A | AGTGGTTGAGCTGGTTGAGG |  |
| IFN-γ-S | GCTGATGGCAATCCTGTTTT | 247 |
| IFN-γ-A | GGATTTTCAAGCCAGTCAGC |  |
| IL-2-S | TCCCTGAATTTCGCCAAG | 156 |
| IL-2-A | AGCGGACAGCAAGTTAGGTAGC |  |
| IL-6-S | TTCGACGAGGAGAAATGCTT | 150 |
| IL-6-A | CCTTATCGTCGTTGCCAGAT |  |
| IL-1β-S | TCATCTTCTACCGCCTGGAC | 149 |
| IL-1β-A | GTAGGTGGCGATGTTGACCT |  |
| MX-S | CGTGATCCGATTGGTTGAA | 218 |
| MX-A | CTGCCTTGGTAGAGCGTAA |  |
| OAS-S | TCTTCCTCAGCTGCTTCTCC | 187 |
| OAS-A | ACTTCGATGGACTCGCTGTT |  |
| PKR-S | AATTCCTTGCCTTTTCATTCAA | 118 |
| PKR-A | TTTGTTTTGTGCCATATCTTGG |  |
| IFIT5-S | AAGAACCCAACAACCCG | 163 |
| IFIT5-A | GCAAGTAGTGCCATAACAACAG |  |
| β-actin-S | GGTATCGGCAGCAGTCTTA | 158 |
| β-actin-A | TTCACAGAGGCGAGTAACTT |  |
| DHAV-3 q-PCR-S | CTTGAACGTAATAGAGCTTGG | 147 |
| DHAV-3 q-PCR-A | AGTCTTTTGGTAGAGTCTTAG |  |

**Additional file 1**
