# Supplementary material for: Comparative liver transcriptome analysis in ducklings infected with duck hepatitis A virus 3 (DHAV-3) at 12 and 48 hours post-infection through RNA-seq
Source: Vet Res. 2018 Jun 20;49:52. doi: 10.1186/s13567-018-0545-7 (PMC6011267; doi:10.1186/s13567-018-0545-7)
Supplement: Supplementary file 2 — Additional file 2. Number of reads of all bases detected using RNA-seq in DHAV-3-infected and control ducks. To guarantee ideal results for genomic mapping and differential gene change analysis, raw reads were filtered to remove low quality data with a total of 437 million (437 610 926) clean reads acquired. [file 13567_2018_545_MOESM2_ESM.docx]

**Additional file 2**

| Library | Number of raw reads | Number of clean reads | Number of uniquely mapped reads | Percentage of reads mapped (%) |
| --- | --- | --- | --- | --- |
| C-1 | 46183094 | 44469670 | 29957023 | 67.37 |
| C-2 | 49552070 | 47880904 | 33631862 | 70.24 |
| C-3 | 51199820 | 49383318 | 35406607 | 71.70 |
| P-12-1 | 44642960 | 43067330 | 30751039 | 70.40 |
| P-12-2 | 53405640 | 51570806 | 35154231 | 68.17 |
| P-12-3 | 48362274 | 45669172 | 31338734 | 68.62 |
| P-48-1 | 56018978 | 53280796 | 37722016 | 70.80 |
| P-48-2 | 55065078 | 52159316 | 35152444 | 67.39 |
| P-48-3 | 53336656 | 50129614 | 33801134 | 67.43 |
| Total | 457766570 | 437610926 | 302915090 |  |
